# Bridging theory and behavior for healthcare accessibility modeling: A mobility-driven revision of the E2SFCA

**DOI:** 10.1371/journal.pone.0346009

**Published:** 2026-04-07

**Authors:** Ali Khosravi Kazazi, Zhenlong Li

**Affiliations:** Geoinformation and Big Data Research Lab, Department of Geography, The Pennsylvania State University, University Park, Pennsylvania, United States of America; King Abdulaziz University, SAUDI ARABIA

## Abstract

Proximity to a healthcare supplier does not necessarily equate to meaningful access to care. Traditional healthcare accessibility models, particularly the Enhanced Two-Step Floating Catchment Area (E2SFCA) method, rely heavily on assumptions of proximity-driven behavior, fixed catchment sizes, and uniform distance decay. These simplifications often overlook the complexities of real-world healthcare-seeking behavior. This study examines the assumptions of the E2SFCA framework by integrating large-scale human mobility data in 2023, which captures anonymized, real-world visitation patterns between neighborhoods and hospitals across Pennsylvania. We revise the E2SFCA model through two key innovations: 1) replacing static catchment thresholds with dynamic, visit-weighted boundaries derived from observed travel behavior, and 2) estimating hospital-specific distance decay functions that better reflect heterogeneous patterns of attraction. These refinements result in accessibility metrics that align more closely with empirical realities. Compared to the traditional model, the revised E2SFCA demonstrates a more meaningful relationship with real-world health outcomes. Specifically, the revised model shows a stronger and statistically significant correlation with household income (r = 0.31, p = 0.011, vs. r = 0.14 in the traditional model) and a more plausible negative association with poor or fair health status (r = −0.12 vs. r = 0.17), aligning with the expectation that better accessibility corresponds to better health outcomes. Additionally, the revised model reveals significantly greater inequality in access that exposes disparities in healthcare accessibility that distance centric approaches tend to obscure. By integrating human mobility data in spatial accessibility modeling, this study offers a more realistic, equitable, and policy-relevant framework for evaluating healthcare access.

## 1. Introduction

Accessibility, defined as the ease of reaching services from a location, is critical for achieving equitable public health outcomes [1]. The World Health Organization (WHO) highlights healthcare accessibility as a key factor in reducing disparities [2], requiring accurate measurement to identify underserved populations. Researchers have developed diverse methods to evaluate access, ranging from simple metrics like provider-to-patient ratios to advanced models simulating individual rational choices [3]. These efforts also distinguish between different types of accessibility, such as objective measures and subjective, perception-based experiences, while acknowledging barriers including socioeconomic disadvantage, transportation gaps, and systemic inequities [4,5].

A widely used approach for measuring healthcare accessibility is the Floating Catchment Area (FCA) method, which aims to balance conceptual simplicity and practical implementation. The Enhanced Two-Step Floating Catchment Area (E2SFCA) method [6] builds on earlier models by incorporating supply, demand, and distance decay, which is the principle that the likelihood of visiting a provider decreases as travel distance increases. While E2SFCA has proven effective in identifying underserved areas, it also has notable limitations. First, it relies on fixed distance thresholds to define catchment areas, leading to abrupt discontinuities in accessibility scores. For instance, a resident living just beyond a mile threshold may be excluded from accessing a nearby supplier, despite its practical proximity. Second, the method is susceptible to edge effects, potentially misclassifying individuals near geographic boundaries if nearby healthcare facilities fall outside the defined catchment. Third, it assumes uniform travel behavior across populations, overlooking important variations such as rural residents who typically travel longer distances, or individuals with disabilities who may face greater mobility constraints [7]. The model also assumes people prioritize proximity, which may oversimplify real-world healthcare decisions. Patients often bypass closer facilities due to insurance networks, referrals, or preferences for specialized care [8]. For instance, a cancer patient might travel long distances to a specialty hospital, defying the model’s assumptions.

To address these gaps, scholars such as Wang (2012) advocate for grounding accessibility models in empirical data [1]. Emerging mobility datasets such as anonymized SafeGraph mobile location data (now Advan patterns) provide valuable insights into actual travel behavior. SafeGraph aggregates visits to facilities from millions of mobile devices, capturing place visitation patterns such as cross-regional healthcare utilization [9]. While challenges remain such as sampling biases that may underrepresent low-income or older populations [10], such data help bridge the gap between theoretical models and real-world dynamics

This study aims to address the limitations of conventional FCA methods by integrating population mobility data into the E2SFCA framework. We revise catchment areas based on observed visitation patterns, replacing fixed distance thresholds with dynamic boundaries that reflect actual patient travel behaviors. Additionally, we refine the distance decay component by deriving hospital-specific decay functions from empirical data, recognizing, for example, that a regional trauma center may draw patients from greater distances, while a neighborhood hospital serves a more localized population. These enhancements are synthesized into a revised E2SFCA model that evaluates healthcare accessibility through a dual lens: one rooted in spatial theory, the other grounded in real-world mobility patterns.

## 2. Related work

Access to healthcare is broadly defined as the “fit” between patient needs and the availability of services [11]. Seminal frameworks describe multiple dimensions of access, including availability, accessibility (geographic), affordability, accommodation, and acceptability, that together determine how well services match patient needs [12]. Levesque et al. (2013) similarly conceptualized access through five abilities of populations to seek care: approachability (awareness of services), acceptability (cultural/social fit), availability and accommodation (physical reach and organization), affordability (economic cost), and appropriateness (fit of services to needs) [13]. These models highlight that spatial barriers (distance, travel time), financial barriers (cost, insurance), and perceived barriers (trust, cultural congruence, knowledge) all influence access. In sum, healthcare accessibility is multidimensional, reflecting service supply relative to demand as well as personal, social, and economic factors.

Early measures of geographic access were relatively simple, often relying on provider-to-population ratios- such as the number of providers per capita in an area, were widely used (e.g., U.S. HPSA scores) [14]. Distance or travel-time measures (e.g., to nearest provider) also became common. However, these one-dimensional metrics ignore spatial distribution of supply and demand. To capture both, gravity-based models were introduced. Joseph & Bantock (1982) proposed an accessibility model that integrates provider capacity and population demand with a distance-decay function [15]. Building on this, Luo and Wang (2003) developed the Two-Step Floating Catchment Area (2SFCA) method: first computing a provider-to-population ratio within a travel-time catchment around each provider, then summing these ratios for each population location’s catchment [16]. The 2SFCA thus yields a local supply-demand ratio that accounts for overlapping service areas. Numerous enhancements followed. Luo & Qi (2009) introduced the Enhanced 2SFCA (E2SFCA) by applying distance-decay weights within catchments, giving nearer populations more weight [6]. Other studies have implemented continuous distance-decay kernels, variable catchment sizes, multi-modal travel, and other refinements [17–19]. These evolving methods, from simple ratios and gravity models to floating catchment approaches (e.g., 2SFCA, E2SFCA), aim to better represent the geographic reality of healthcare access.

Despite its advances, the E2SFCA (and related FCA) framework has known limitations. (1) Fixed catchment sizes: Standard 2SFCA/E2SFCA typically use one travel-time radius (e.g., 30–60 minutes) for all locations. This ignores that urban populations may accept short travel while rural populations tolerate longer trips. (2) Edge effects: Restricting analysis to administrative boundaries can bias results because people near borders may use providers across the border, but these are excluded, leading to artificial “access deserts” [14]. (3) Uniform travel assumptions: These methods assume homogeneous travel behavior (same speed, mode) for all individuals. In reality, mobility varies by age, car ownership, disability, etc., which the models do not capture. (4) Dichotomous accessibility: Traditional 2SFCA (and even E2SFCA with stepwise weights) treats providers within the threshold as fully accessible and those outside as inaccessible [6]. This sharp cut-off is unrealistic because in reality access declines gradually with distance. (5) Proximity prioritization: Implicitly, these models assume individuals use the nearest available providers first, filling up capacity in distance order, which may not reflect personal preferences or provider choice behavior. (6) Overcounting demand: By assigning full population demand to every facility within range, 2SFCA can overestimate demand if individuals are counted at multiple providers simultaneously. In short, E2SFCA simplifies human behavior and spatial context (fixed zones, equal weights), leading to edge effects and untested assumptions about travel and choice [14].

Recent years have seen growing use of real-world mobility data to study healthcare and service access. Data sources include anonymized mobile-phone location datasets (SafeGraph, Cuebiq, Google, etc.) and call detail records (CDRs) from telecom providers. These capture where people actually travel and which facilities they visit. For example, Wang et al. (2021) used SafeGraph data to analyze patterns of healthcare visits during the COVID-19 pandemic [20]. They clustered census block groups by temporal trends in visits and linked declines to demographic factors, illustrating how mobility data reveal access disparities in practice. More broadly, Swanson & Guikema (2024) developed methods using cellphone “location-based services” data to assess community-level loss of access to essential services (including healthcare) after disasters [21]. These studies demonstrate strengths of mobility data: large-scale, high-frequency observations of actual travel behavior and utilization.

A few emerging studies have begun to integrate mobility into spatial accessibility models. Chen et al. (2024) propose a Generalized Flow-based 2SFCA (GF2SFCA) that explicitly uses observed patient flows (from mobility data) to inform the model [22]. In GF2SFCA, hospital catchment sizes, distance-decay rates, and attractiveness weights are data-driven: for example, hospitals’ “global popularity” and “local preference” indices are computed from actual visitation counts and travel distances. In a Wuhan case study, this approach produced more realistic accessibility maps than conventional 2SFCA and showed robustness to data uncertainties [22]. Though focused on hospital access, the principle applies more broadly. Other work (e.g., in urban parks and food access) similarly uses travel-behavior data to adjust catchment radius and decay functions. Overall, these hybrid models improve realism by calibrating FCA parameters with real mobility, moving beyond arbitrary assumptions.

The literature reveals at least two notable gaps in current healthcare accessibility research. First, the integration of actual mobility data into FCA models remains limited. While recent methodological advances like the GF2SFCA [22] have been proposed, hybrid approaches that combine empirical travel data with FCA methods are still rare. Second, there is a lack of systematic validation of the core assumptions underlying the E2SFCA model, such as fixed travel thresholds, uniform distance decay, and proximity-based behavior, against observed patient travel patterns. Researchers have pointed out that the scarcity of patient travel data constrains this validation [14]. Consequently, the behavioral assumptions foundational to E2SFCA remain largely untested and unverified in empirical research.

The literature also highlights the importance of grounding healthcare accessibility models in actual mobility patterns. In the context of Pennsylvania, prior research illustrates the stakes: Drake et al. (2021) found that basic measures such as straight-line distance or provider-to-population ratios failed to identify 30–52% of census tracts that the E2SFCA model classified as underserved [7], emphasizing the need for more sophisticated spatial approaches. Building on these insights, our study employs SafeGraph mobility data to empirically revise key E2SFCA parameters. Specifically, we analyze observed visit flows to Pennsylvania hospitals to define catchment boundaries and assign distance decay weights that reflect real-world travel behavior. This integration directly addresses known limitations of the E2SFCA model, for example, replacing uniform catchment thresholds with dynamic radius that reflect actual willingness to travel, and tailoring decay functions to match empirically observed declines in visitation with distance. The resulting model, which integrates the theoretical strengths of FCA accessibility with empirical mobility data, represents a novel contribution.

While recent advancements have successfully integrated mobility data into accessibility modeling, our proposed framework diverges in its model formulation and parameter calibration strategy. Chen et al. (2024) utilize taxi trace data to construct “Global Popularity” and “Local Preference” indices, embedding them into a Huff-model probability framework to simulate hospital attractiveness [22]. In contrast, our mobility-driven revision retains the intuitive structure of the E2SFCA but directly replaces its arbitrary parameters with metrics derived from broader human mobility data. Specifically, rather than modeling selection probabilities via popularity indices, we introduce the visit-weighted average distance to empirically define dynamic catchment boundaries. This ensures that the spatial extent of the model reflects the actual distance patients are willing to travel, rather than a theoretical probability of selection.

Furthermore, this work distinguishes itself from earlier variable-catchment and modified models such as the V2SFCA and M2SFCA by prioritizing observed behavior over theoretical optimization. The V2SFCA [23] varies catchment sizes dynamically, but does so based on a theoretical “optimization” process which expands catchments incrementally until a target base population or provider-to-population ratio is met. Our model, conversely, defines variable catchments based on empirical visitation footprints, capturing the heterogeneity of rural versus urban travel behavior as it actually occurs. Similarly, while the M2SFCA [24] mathematically adjusts the FCA metric to account for suboptimal system configurations (absolute distance impedance), it typically relies on standard distance decay functions. Our approach advances this by empirically fitting hospital-specific decay functions (e.g., power-law vs. linear) to observed flows, thereby calibrating the model with the specific spatial attenuation patterns of each facility rather than a generalized system-wide adjustment.

## 3. Methodology

This study systematically evaluates the core assumptions of the E2SFCA method by comparing them against empirical human mobility patterns to assess their validity in real-world context. The E2SFCA method is built on four main assumptions: 1) both suppliers and demanders operate within bounded geographic catchment areas [6]; 2) these catchments are fixed and of equal size across all locations [25]; 3) within these catchments, individuals’ willingness to access services decreases with increasing distance (distance decay) [24]; and 4) this distance decay is identical for all suppliers and demanders, regardless of local variation [26]. For each assumption, we follow a two-step process. First, we evaluate its alignment with empirical data by comparing the theoretical premise to observed mobility patterns, including visitation flows and distance decay trends. Second, based on this assessment, we either refine or revise the assumption as needed. If an assumption aligns with empirical evidence, we enhance its precision (e.g., replacing fixed catchment sizes with thresholds derived from observed mobility data). If it diverges from observed behavior, we discard or reformulate it (e.g., substituting uniform distance decay with variable, context-specific functions). By anchoring each assumption in real-world data, this study bridges the gap between spatial accessibility theory and observed healthcare-seeking behavior.

### 3.1. Data and study setting

This study focuses on Pennsylvania, U.S. The state’s geographic diversity introduces substantial variation in healthcare infrastructure and physical accessibility, shaped by natural barriers, population density gradients, and uneven hospital distribution. These spatial disparities intersect with demographic and structural inequities: rural communities face aging populations and mobility constraints, while wealth disparities and environmental health risks disproportionately burden underserved urban and peri-urban neighborhoods. Together, these dynamics make Pennsylvania a compelling case study for analyzing spatial accessibility in a way that reflects broader national trends.

The analysis integrates multiple data sources across three spatial scales: block groups (n = 9,740), counties (n = 67), and hospitals (n = 191). Demographic and socioeconomic data, including population and mean household income (S1 File), were obtained from the U.S. Census Bureau’s American Survey (ACS) 5-Year Estimates for 2018–2022. These data provide the demand-side context for block groups and support county-level aggregation for validation against public health outcomes [27]. Hospital data were acquired from the Commonwealth of Pennsylvania Department of Health (S2 File), including facility names, addresses, and the number of licensed beds as a proxy for service capacity [28]. The dataset encompasses a full spectrum of hospital types, including general acute care, specialty, and federal hospitals. Health outcome data, specifically the percentage of the population reporting poor or fair health, were sourced from the County Health Rankings and Roadmaps (S3 File) [29], allowing for validation of accessibility scores at the county level.

To capture empirical travel behavior, the study utilizes SafeGraph’s (now Advan patterns) anonymized human mobility dataset (S4 File), which includes over 3.5 million hospital visits across Pennsylvania during calendar year 2023 [30]. Visits were aggregated at the census block group level and linked to hospitals using address matching. To accurately link mobility visits to hospitals, we implemented a structured address-matching process. The hospital dataset provided facility names and county information, which we used to query the Google Maps API and obtain standardized addresses. We then used the Placekey platform [31] to find the unique Placekey for each hospital, which separates spatial location (“where”) from descriptive attributes (“what”). To ensure consistent spatial referencing, we extracted the “where” component of each Placekey and compared it to those in the mobility dataset. For each match, we applied fuzzy string matching [32] to hospital names to identify the most likely corresponding record. This approach enabled reliable linkage between hospital locations and mobility data, even when address formats or facility names varied slightly. In total, 191 hospitals were successfully matched and included in the final analysis. Geodesic distances, measured in miles between centroids of origins and destinations, were used to estimate travel distances in all modeling steps.

### 3.2. Evaluate the bounded catchment areas assumption

A central methodological debate in spatial accessibility modeling concerns whether to constrain patient–provider interactions within predefined catchment areas or to adopt an open, boundary-free approach. Catchment-based models, such as the E2SFCA, impose geographic limits (typically based on travel time or distance), around healthcare providers and their surrounding populations. While this structure simplifies computation and aligns with administrative planning, it risks oversimplifying real-world mobility, as patients may bypass nearby facilities in favor of more specialized or preferred providers farther away. In contrast, open models remove arbitrary thresholds and can capture long-distance travel patterns, but they risk overestimating accessibility by assuming that all populations, including those in remote areas, have equal access to distant hospitals, potentially introducing noise from unrealistic interactions.

This assumption test empirically evaluates whether catchment areas reflect actual human mobility behaviors and seeks to clarify whether spatial accessibility frameworks should impose bounded catchment areas or adopt a more flexible, boundary-free approach grounded in empirical mobility data.

From the supplier (hospital) perspective, we compute:

1 Hospital coverage ratio:

$${HosCovRatio}_{j}=\frac{N_{j}}{N_{BG}}$$

where $N_{j}$ is the number of unique block groups visiting hospital j and $N_{BG}$ is the total number of block groups.

2 Relative visit-weighted distance for hospitals (HosRWD)

$${\text{HosRWD}}_{j}=\;\frac{\sum_{i\in V_{j}}w_{ij}\;.\;d_{ij}}{\sum_{i\in V_{j}}w_{ij}}\;÷\;\frac{\sum_{i=\text{1}}^{N_{BG}}d_{ij}}{N_{BG}}$$

where $w_{ij}$ is the number of visits from block group i to hospital j, $d_{ij}$ is the distance between them and $V_{j}$ is set of block groups with non-zero visits to hospital j.

From the demand (population at block group level) perspective, we compute:

1 Hospital Choice Ratio (PopChoiceRatio)

$${\text{PopChoiceRatio}}_{i}=\frac{M_{i}}{N_{Hosp}}$$

where $M_{i}$ is the number of unique hospitals visited by block group i and $N_{Hosp}$ is the total number of hospitals

2 Relative visit-weighted distance for block groups (PopRWD)

$${\text{PopRWD}}_{i}=\;\frac{\sum_{j\in V_{i}}w_{ij}\;.\;d_{ij}}{\sum_{j\in i}w_{ij}}\;÷\;\frac{\sum_{j=\text{1}}^{N_{Hosp}}d_{ij}}{N_{Hosp}}$$

where $V_{i}$ is set of hospitals visited by block group i.

Lower values in these metrics (especially the relative distance ratios) indicate more localized, catchment-like behavior, supporting the use of geographic boundaries in accessibility models. Higher values suggest broader or more dispersed travel behavior, consistent with an open, boundary-free model.

### 3.3. Evaluate the fixed catchment sizes assumption

A key critique of fixed catchment sizes is their failure to account for variations in travel behavior, particularly the tendency of rural populations to travel longer distances for care compared to urban residents. Numerous studies argue that catchment boundaries should reflect observed mobility patterns rather than rely on arbitrary thresholds. To address this limitation, our study introduces a visit-weighted average distance metric for both population origins and hospital destinations.

For each block group i, the visit-weighted average distance to utilized hospitals is calculated as:


$${VWAD}_{i}=\;\frac{\sum_{j\in V_{i}}w_{ij}\;.\;d_{ij}}{\sum_{j\in i}w_{ij}}$$

where $w_{ij}$ is the number of visits from block group i to hospital j, and $d_{ij}$ is the distance between them.

Similarly for each hospital j the average distance from its visitors is:


$${VWAD}_{j}=\;\frac{\sum_{i\in j}w_{ij}\;.\;d_{ij}}{\sum_{i\in j}w_{ij}}$$

This metric captures how far individuals actually travel by weighting each distance according to the number of visits to a given hospital.

This approach offers three distinct advantages. First, it enhances behavioral realism by prioritizing observed travel behavior over theoretical assumptions. Second, it incorporates visit intensity, ensuring that hospitals frequently visited by patients exert a proportionally greater influence on the calculated average, thus capturing disparities in healthcare utilization. Third, it accounts for heterogeneity in both provider and population behavior, recognizing that certain hospitals (e.g., specialized or regional centers) may attract patients from greater distances, while some communities routinely travel farther for care.

### 3.4. Distance decay consideration

The E2SFCA method incorporates distance decay in two sequential steps: 1) hospitals attract fewer visitors as the distance to surrounding neighborhoods increases, and 2) residents reduce their utilization of hospitals as travel distance grows. This dual-decay framework assumes a symmetrical decline in interaction likelihood between supply and demand as spatial separation increases. To evaluate this assumption, we fit both power-law and linear (negative exponential in log-linear form) distance decay models to empirical hospital–neighborhood visit data. The power-law model takes the form $\omega_{ij}=\;\alpha \overset{-\beta }{\underset{ij}{d}}$ while the exponential model is expressed as $\omega_{ij}=\alpha e^{-\beta d_{ij}}$. For each hospital, we estimated model parameters and assessed fit quality using two criteria: coefficient of determination (R²) and Akaike Information Criterion (AIC). We then classified the best-fitting model using a systematic rule-based approach.

A hospital was classified as following a power-law decay if the power-law model outperformed the linear model on both R² and AIC metrics, and its distance coefficient was significantly negative (β < 0, p < 0.05). Conversely, it was classified as following a linear decay if the linear model showed superior fit by the same criteria and also had a significantly negative coefficient. If neither model met these conditions, i.e., if the better-fitting model lacked a statistically significant negative coefficient, the hospital was categorized as exhibiting no clear decay.

### 3.5. Evaluate the uniform distance decay assumption

The E2SFCA method assumes a uniform distance decay function applies identically to all suppliers and neighbors, disregarding potential variability in spatial interaction behavior. To test this assumption, we estimate hospital-specific decay parameters using a power-law model ($W(d)=\;d^{-\beta }$). For each hospital, we fit the model to its visitation data to derive a unique decay parameter (β), reflecting how rapidly visitation frequency declines with distance. This approach allows us to quantify heterogeneity through comparing decay rates across suppliers and evaluate uniformity to assess whether a single decay parameter is sufficient. This test is expected to provide new insights on whether the uniform decay assumption aligns with empirical patterns or obscures critical variations in healthcare-seeking behavior, advocating for context-sensitive decay functions in spatial accessibility modeling.

### 3.6. Development of revised method

Following the independent evaluation of the four core assumptions underlying the E2SFCA method, we develop a revised spatial accessibility framework (detailed in Section 4.5) that integrates the methodological insights derived from the preceding analyses. The proposed framework incorporates key modifications to the original E2SFCA structure. These adjustments are designed to enhance the model’s capacity to represent observed patterns of healthcare-seeking behavior and spatial interaction.

## 4. Results and discussion

The results highlight how traditional spatial accessibility metrics that are rooted in assumptions of distance minimizing behavior and uniform distance decay can misrepresent actual patterns of healthcare utilization. By recalibrating the E2SFCA model using empirically derived dynamic catchment areas and variable distance decay parameters, we reveal more context-sensitive disparities in spatial access. These findings demonstrate that mobility-informed refinements yield a more realistic and equitable representation of healthcare accessibility.

### 4.1. Bounded catchment areas

This section empirically evaluates a core assumption underpinning spatial accessibility models like the E2SFCA method: that healthcare interactions are geographically constrained and typically occur within bounded catchment areas. While this assumption is often taken for granted in the design of such models, few studies have systematically quantified the extent to which real-world healthcare interactions align with this theoretical framework.

As shown in Table 1, our analysis reveals a pronounced localization in hospital–patient interactions. From the hospital perspective, the average visit-weighted distance to patients (HosRWD) is only 33.53 miles, in contrast to the 130.9-mile average distance across all block groups. On average, each hospital serves only 340 out of 9,740 block groups, or about 3.5% of the possible population base. This indicates a sharply bounded spatial reach for most hospitals. From the block group perspective, the average visit-weighted distance to utilized hospitals (PopRWD) is just 14.7 miles, compared to 130.9 miles if all hospitals were considered equally. Furthermore, each block group engages with only 5 out of 191 hospitals on average (2.6%), reinforcing the conclusion that residents overwhelmingly seek care from a small, nearby subset of providers.

**Table 1:** Summary of interaction patterns between hospitals and block groups.

Perspective	Metric	Value
Hospital	Avg. visit-weighted distance visitors to hospital	33.53 miles
	Avg. distance to all block groups (mean of means)	130.9 miles
	Avg. number of block groups served per hospital	340
	Total number of block groups	9,740
	Avg. visit-weighted distance to utilized hospitals	14.7 miles
Block Group	Avg. number of hospitals visited per block group	5
	Avg. distance to all hospitals (mean of means)	130.9 miles
	Total number of hospitals	191

Although the original E2SFCA method implicitly assumes localized interactions via predefined catchments and distance decay functions, it does not specify whether these assumptions match empirical behavior. The significant differences between actual travel distances and theoretical full-network distances validate that spatial interaction is indeed bounded in practice. This spatial concentration of interactions justifies the continued use of catchment areas in spatial accessibility models.

### 4.2. Fixed catchment sizes

While the previous section demonstrated that patient-provider interactions are geographically bounded, this section focuses on how those bounds are represented in accessibility models. Specifically, it critiques the common use of fixed-distance catchment sizes, showing that actual travel behavior varies significantly across contexts and cannot be captured by a one-size-fits-all threshold.

To more accurately reflect real-world travel behavior, we employed a visit-weighted average distance metric, which adjusts travel distances based on the frequency of visits to each hospital. Unlike arbitrary static thresholds (e.g., a fixed 30-mile catchment), this metric captures the actual spatial extent of interactions, offering a more behaviorally grounded approach to defining access. The average VWAD for all hospitals was 33.5 miles (median = 28.9 miles), while residents traveled an average of 14.7 miles (median = 11.14 miles) to access care. These findings suggest that a one-size-fits-all catchment, such as 30 miles, may either overestimate or underestimate access depending on local conditions, provider type, and population characteristics.

**Fig 1:**
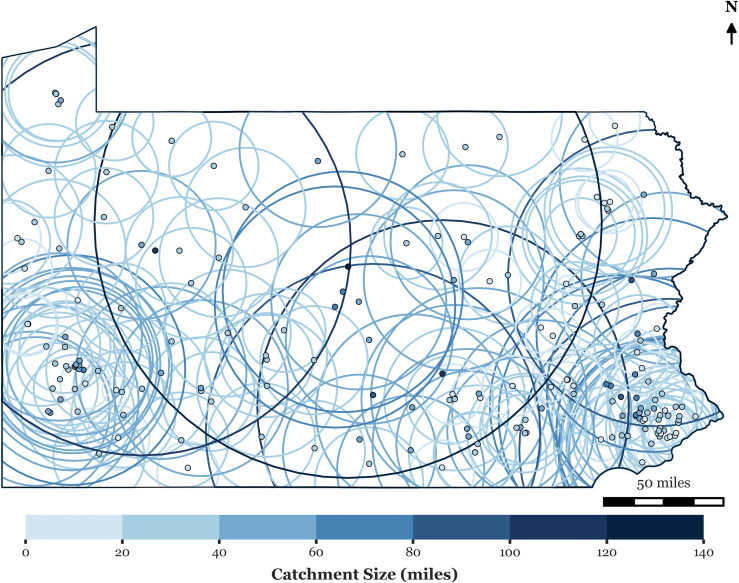
Hospitals’ dynamic catchment area.

**Fig 2:**
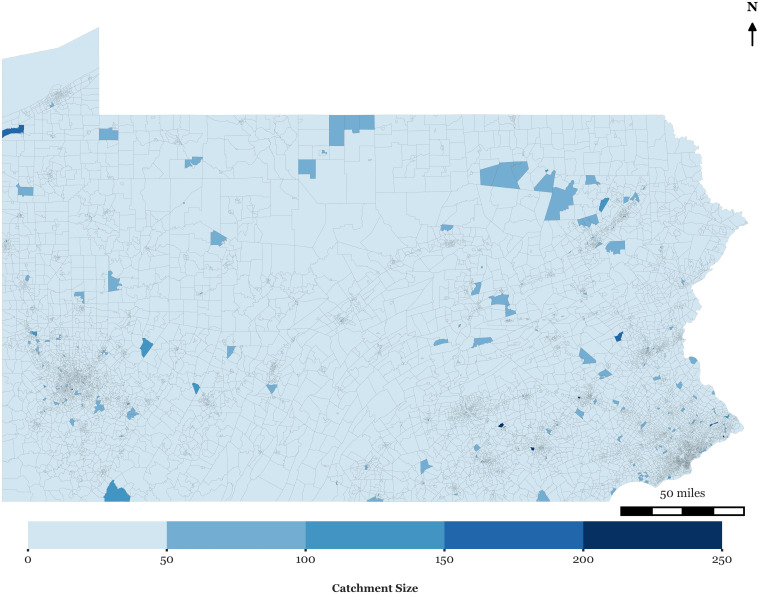
Block groups’ dynamic catchment sizes.

Fig 1 illustrates the variability of catchment sizes by mapping dynamic catchment areas for hospitals, where the spatial reach expands or contracts based on actual visitation patterns. Fig 2 shows catchment sizes for block groups. The underlying data reveal similarly uneven patterns of hospital utilization. These findings suggest that visit-weighted distance metrics offer a more flexible and empirically grounded alternative to fixed catchment sizes, allowing spatial accessibility models to better reflect how far people actually travel for care.

### 4.3. Distance decay consideration

The analysis of distance decay is critical for understanding how spatial friction influences healthcare interactions. To capture this empirically, we evaluated the visitation patterns for all hospitals in the study area. Using a rigorous model selection process governed by the AIC and the coefficient of determination (R²), we assessed whether hospital-specific distance decay behavior was best described by a power-law or an exponential function. A threshold of ΔAIC> 2 was utilized to indicate a meaningful statistical improvement of one model over the other.

The calibration results reveal significant heterogeneity in healthcare-seeking behavior across the modeled hospitals. As summarized in Table 2, the power-law model provided a superior fit for the majority of facilities (hospitals, 68.3%). This indicates a heavy penalty for short distances with a long tail, representing steep drop-offs in patient volume beyond the immediate neighborhoods surrounding the hospital. Conversely, the exponential function was the optimal fit for 31.7% of hospitals, representing a smoother, more gradual decline in visitation over distance. Across all facilities, the empirical fitting yielded an average R² of 0.331 and a median β parameter of –0.26 (S5 Table).

**Table 2:** Summary of distance decay model calibration.

Metric	Value
Hospitals favoring Power-Law (ΔAIC > 2)	68.3%
Hospitals favoring Exponential (ΔAIC > 2)	31.7%
Average Model R²	0.331
Median β Estimate	–0.26

**Fig 3:**
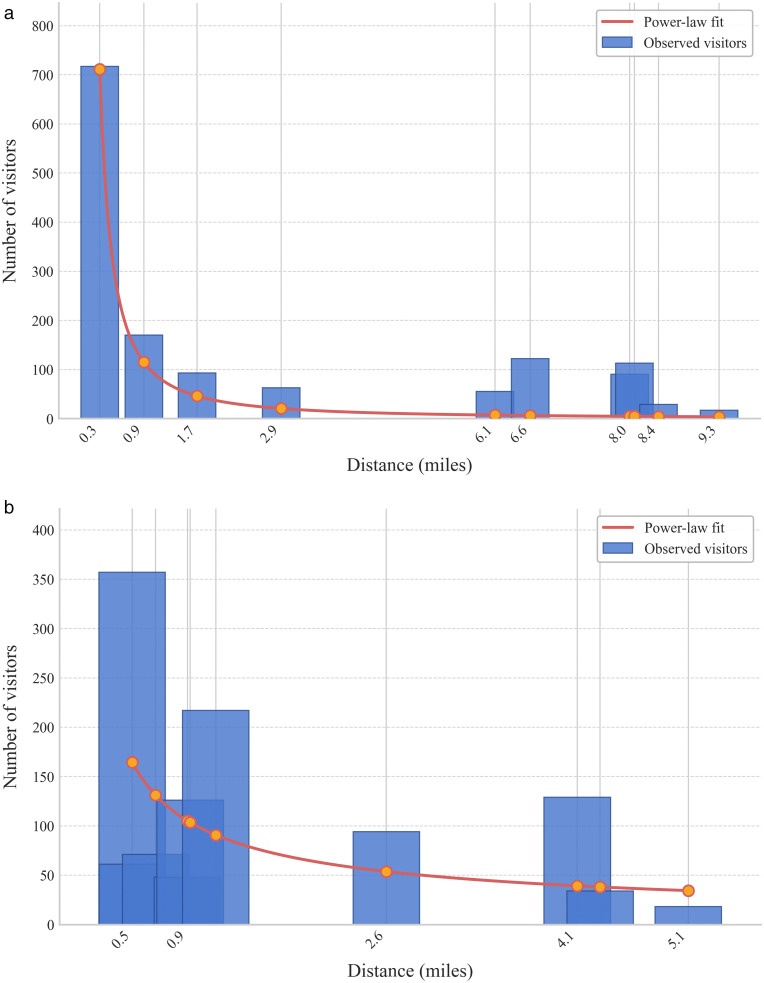
Two samples of the distribution of visits over distance comparing the fitted power-law and exponential models. a) Example of Power-Law distance decay. b) Example of Exponential distance decay.

Fig 3 illustrates these two distinct distance decay patterns derived from the mobility data. Fig 3a shows a hospital catchment best characterized by a power-law decay, where visitation drops sharply near the facility. Fig 3b depicts a hospital conforming to an exponential decay, where patient origins are dispersed more gradually and evenly across the region.

These findings highlight a critical limitation in traditional E2SFCA applications, which almost universally assume a static, uniform distance decay function (e.g., a single β value or a single functional form applied equally to all facilities). The empirical mobility data proves that hospital catchments are highly heterogeneous. By dynamically assigning the statistically optimal functional form (power-law or exponential) and a unique β parameter to each specific hospital, our mobility-driven framework captures the true complexity of real-world healthcare access far better than static global assumptions.

### 4.4. Uniform distance decay

To evaluate the assumption of uniform distance decay across hospitals, we modeled visitation patterns using a power-law function: $visitors=\;\alpha \;.\;d^{-\beta }$, where α represents baseline visitation (i.e., interaction at short distances), and β denotes the decay rate. Each hospital was fitted with its own set of parameters, allowing us to capture provider-specific distance decay behavior.

The median decay rate (β) across hospitals was –0.26, indicating a relatively gradual decline in visitation with increasing distance for most providers. The mean β, at –0.24, closely aligned with the median, suggesting a relatively symmetric distribution with limited influence from outliers. This pattern points to greater consistency in distance decay behavior across providers than previously observed. In contrast, the baseline visitation parameter (α) exhibited more variation. The median α was 38.8, reflecting strong local interaction for a typical hospital, while the mean α was higher at 56.1, indicating a moderate right-skew driven by hospitals experiencing unusually high visitation from nearby populations. This wide variability in both decay and baseline interaction reveals the limitations of using a uniform distance decay function across all providers. These findings support the need for hospital-specific decay parameters to more accurately reflect the diverse spatial behaviors in healthcare utilization.

### 4.5. Revised E2SFCA method

Building on the empirical evaluation of the four foundational assumptions of the E2SFCA method, we developed and implemented a revised spatial accessibility model that reflects observed patterns in healthcare-seeking behavior. The two steps of the revised method are:

***Step 1:*** Estimate the distance-decay-adjusted supply-to-demand ratio for provider j using an empirically fitted power-law decay function.


$$R_{j}=\;\frac{S_{j}}{\sum_{i\in C_{j}}\alpha_{j}.\;\overset{{-\beta }_{j}}{\underset{ij}{d}}.P_{i}}$$

where the index j uniquely represents a specific healthcare supply location (an individual hospital), and the index i represents a specific population demand location (a Census Block Group). Accordingly, $S_{j}$ represents the supply capacity at hospital j, $p_{i}$ is the population size at location i, and $d_{ij}$ is the network distance between the population at i and the hospital at j. Furthermore, $\alpha_{j}$ and $\beta_{j}$ are the hospital-specific distance decay parameters calibrated from mobility data, and $C_{j}$ is the dynamic catchment area specifically serving hospital j, defined by a radius equal to its Visit-Weighted Average Distance ${VWAD}_{j}$.

***Step 2:*** For each population location i, define its dynamic catchment $C_{j}$, based on its ${VWAD}_{i}$. Then, aggregate all accessible providers within this radius to compute the accessibility score.


$$A_{i}=\;\sum_{j\in C_{i}}R_{j}$$

This section provides a reproducible description of the proposed mobility-driven revision of the E2SFCA method. Building on the empirical tests of the four E2SFCA assumptions, the revised method (i) defines dynamic catchment radii using visit-weighted average travel distance derived from observed visits, and (ii) applies hospital-specific distance-decay functions estimated from visitation flows. The complete computational workflow of the revised E2SFCA method is detailed in Algorithm 1.

**Algorithm 1: Mobility-Driven Revised E2SFCA**

**Input**

B: Set of all population locations (block groups), i ∈ B

H: Set of all healthcare providers (hospitals), j ∈ H

P_i_: Population at location i

S_j_: Supply capacity at provider j

d_ij_: Geographic distance between location i and provider j

w_ij_: Observed visit count from location i to provider j (mobility data)

**Output**

A_i_: Spatial accessibility score for each population location i

**Phase 1: Empirical Parameter Estimation**

For each provider j ∈ H do:

V_j_ ← {i ∈ B | w_ij_ > 0}

VWAD_j_ = (Σ (w_ij_· d_ij_)) / (Σ w_ij_)

Fit power-law decay model:

w_ij_ = α_j_· d_ij_^−β^_j_

Extract hospital-specific parameters: α_j_, β_j_

For each location i ∈ B do:

V_i_ ← {j ∈ H | w_ij_ > 0}

VWAD_i_ = (Σ (w_ij_· d_ij_)) / (Σ w_ij_)

**Phase 2: Step 1 – Compute Provider-to-Population Ratio**

For each provider j ∈ H do:

C_j_ ← {i ∈ B | d_ij_ ≤ VWAD_j_}

WeightedDemand_j_ = 0

For each location i ∈ C_j_ do:

W_ij_ = α_j_· d_ij_^−β^_j_

WeightedDemand_j_ = WeightedDemand_j_ + (P_i_· W_ij_)

R_j_ = S_j_ / WeightedDemand_j_

**Phase 3: Step 2 – Compute Spatial Accessibility**

For each location i ∈ B do:

C_i_ ← {j ∈ H | d_ij_ ≤ VWAD_i_}

A_i_ = 0

For each provider j ∈ C_i_ do:

w_ij_ = α_j_· d_ij_^−β^_j_

A_i_ = A_i_ + R_j_

Return A = {A_i_ | i ∈ B}

### 4.6. Accessibility maps

**Fig 4:**
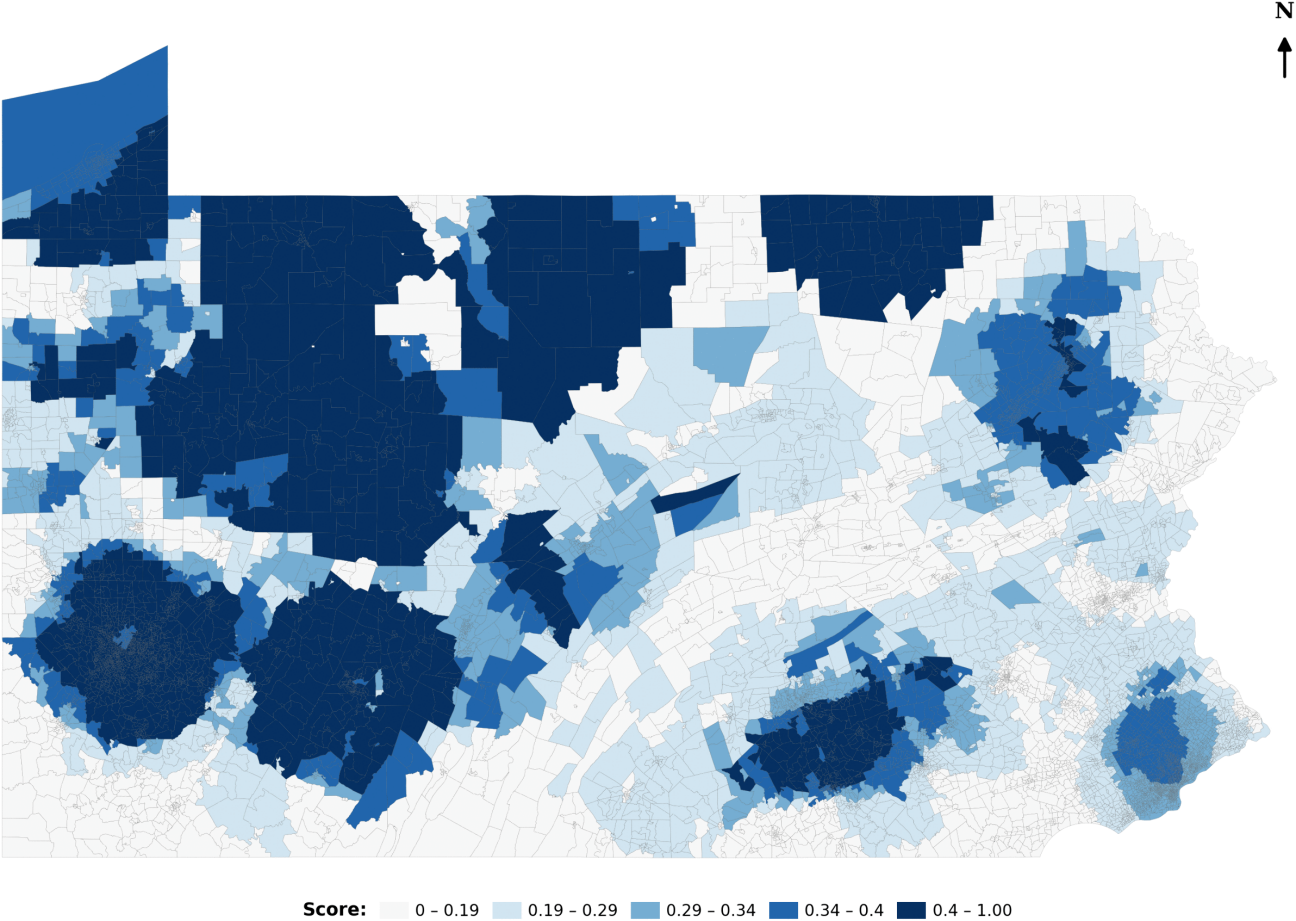
Accessibility map based on E2SFCA and default parameters.

The study concludes with a comparative analysis of healthcare accessibility in Pennsylvania, contrasting the original E2SFCA method with default parameters from Drake et al. (2021) with the revised E2SFCA framework empirically calibrated using human mobility data [7]. To ensure comparability, the accessibility scores from both approaches were normalized using a min–max scaler, rescaling values between 0 and 1. Fig 4 presents accessibility scores derived from the original E2SFCA method. As expected, these scores exhibit a distance decay dominated pattern: neighborhoods located near hospitals receive uniformly high accessibility ratings, while access steadily declines with increasing distance.

**Fig 5:**
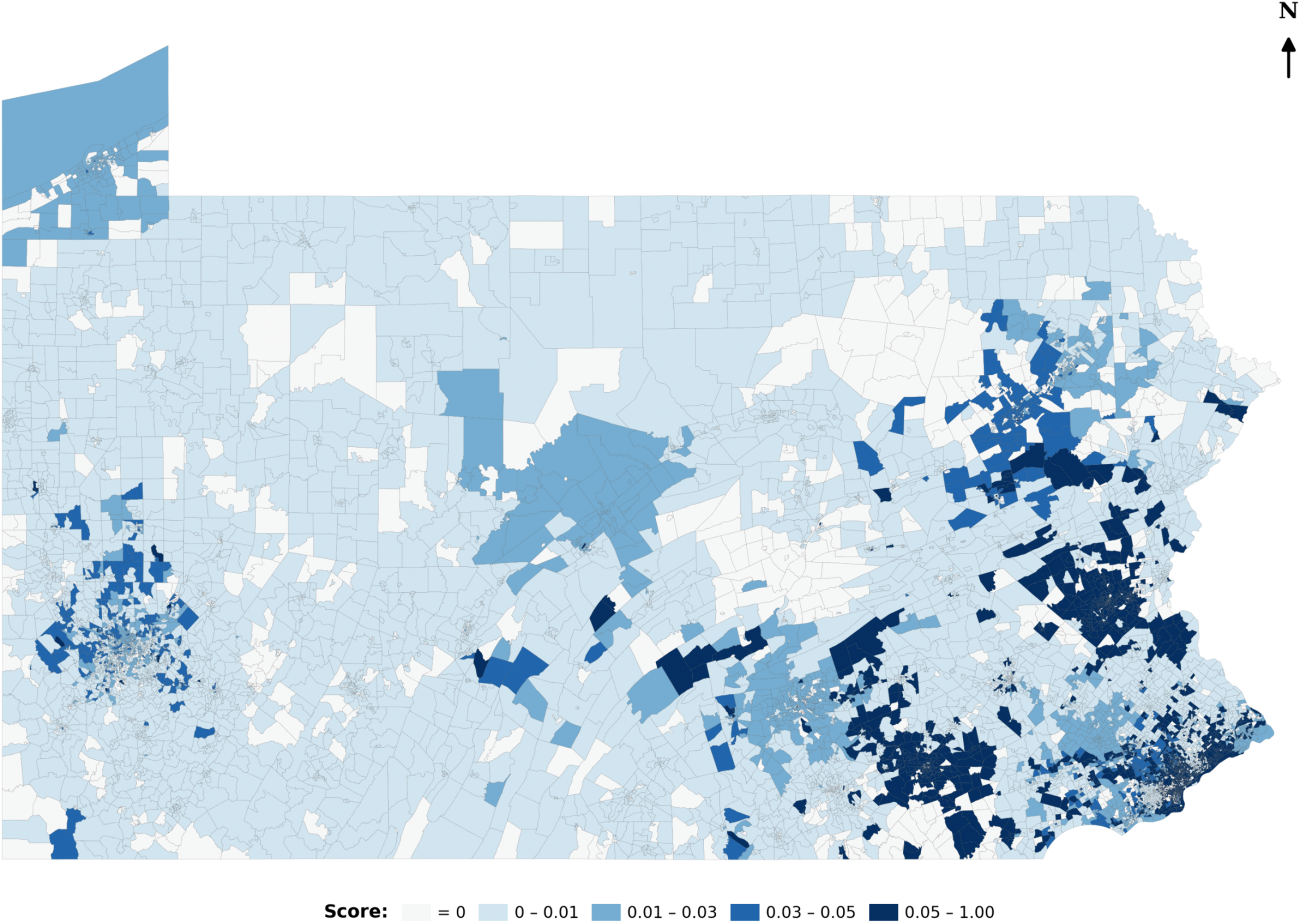
Accessibility map based on revised E2SFCA.

This pattern reflects the underlying assumptions of fixed catchment sizes and uniform distance decay. In contrast, Fig 5 maps accessibility using the revised E2SFCA model, which incorporates dynamic catchment areas, hospital-specific distance decay rates, and empirically derived attractiveness indices. This approach produces a more nuanced accessibility landscape, revealing disparities that are obscured by the traditional model, such as hospitals with high regional pull or underserved areas near densely located but overburdened providers. By visualizing and comparing both approaches, this analysis demonstrates how empirically grounded refinements can improve the realism and equity of spatial accessibility assessments.

Beyond the Pennsylvania case study, this scalable mobility-driven framework empowers policymakers and planners to identify true behavioral healthcare deserts for equitable resource allocation and to optimize dynamic emergency response routing during patient surges. Furthermore, this approach can be adapted to national contexts and other critical infrastructure, ensuring that future spatial planning aligns with actual human behavior rather than rigid geographic assumptions.

## 5. Validation

Given the multifaceted nature of healthcare accessibility, validating spatial accessibility models is inherently challenging. Accessibility is shaped by a complex interplay of geographic, socioeconomic, behavioral, and institutional factors, many of which are not fully captured in spatial models. Considering this complexity, we validate our results through multiple complementary approaches, each offering insight into the realism and performance of the revised E2SFCA method.

### 5.1. Correlation with health outcomes

**Fig 6:**
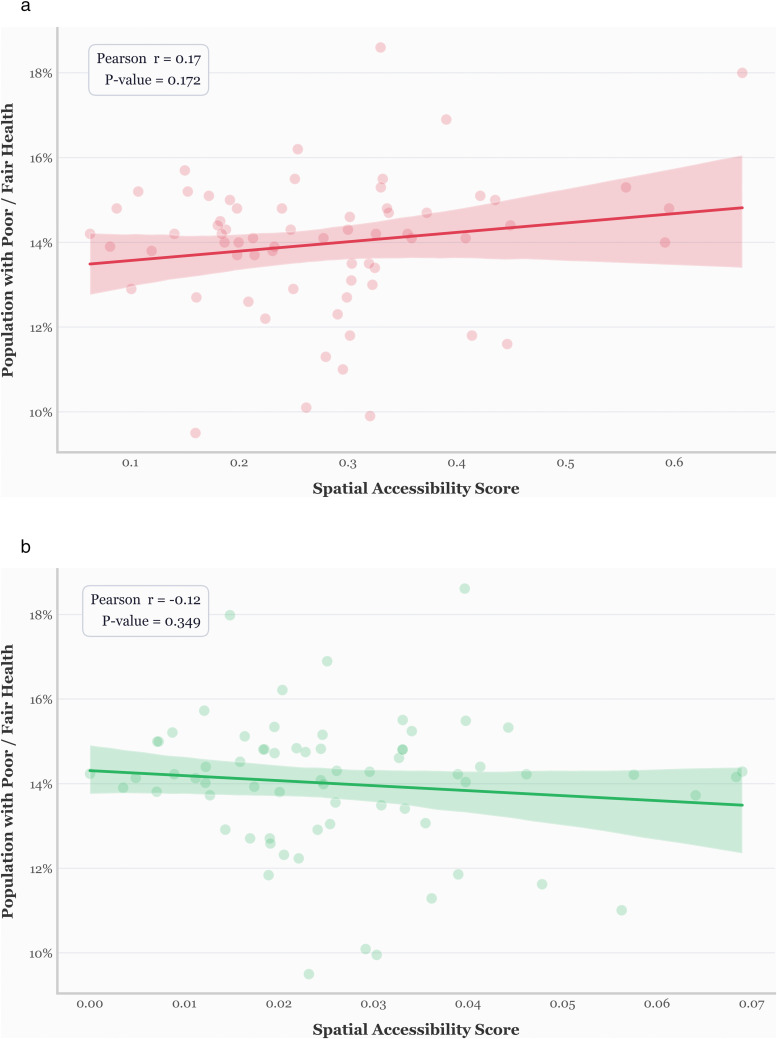
Comparative correlation of traditional and revised accessibility models with health outcomes. a) The E2SFCA with default values. b) Revised E2SFCA.

The first validation compares accessibility scores from both the traditional and revised E2SFCA models to public health outcomes—specifically, the percentage of the population reporting poor or fair health across Pennsylvania counties. Because the health outcome data is available at the county level, we aggregated our block-group-level accessibility scores to match this spatial resolution. Fig 6 shows the association of accessibility models with county-level health status.

As shown in Fig 6a, the traditional E2SFCA method exhibits a weak positive correlation (r = 0.17, p = 0.172) with poor or fair health outcomes, suggesting higher accessibility is paradoxically associated with worse health, which contradicts real-world expectations. In contrast, the revised method (Fig 6b) shows a slightly negative correlation (r = –0.12, p = 0.35), which aligns more plausibly with the expectation that improved accessibility is associated with better health. While neither result is statistically significant, an outcome expected given the many confounding factors influencing health, the revised model demonstrates a directionally more realistic relationship.

### 5.2. Correlation with household income

**Fig 7:**
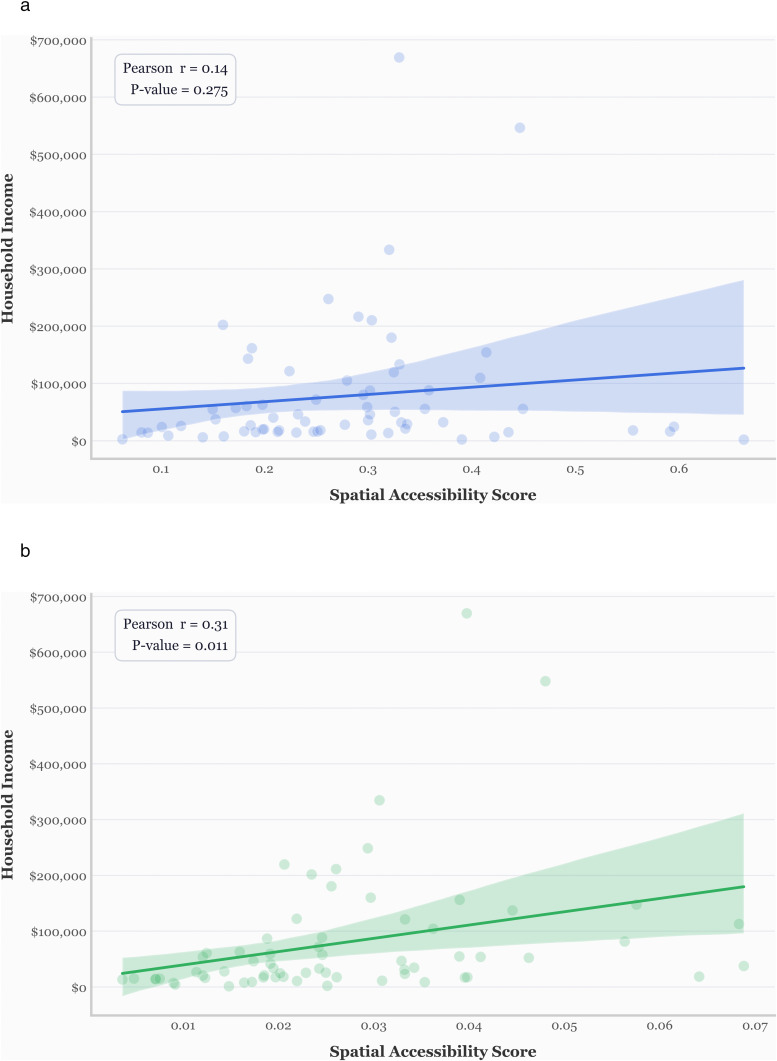
Accessibility and income: validating spatial models against socioeconomic indicators. a) The E2SFCA with default values. b) Revised E2SFCA.

The second validation compares accessibility scores to median household income, under the premise that wealthier populations typically enjoy better spatial access to healthcare [33,34]. Fig 7 represents comparative relationship between accessibility models and household income.

As shown in Fig 7a, the traditional E2SFCA method yields a weak, non-significant correlation (r = 0.14, p = 0.27). However, the revised model (Fig 7b) shows a stronger and significant correlation (r = 0.31, p = 0.011), indicating that the revised scores better reflect socioeconomic patterns known to affect access.

### 5.3. Multivariate Validation and Spatial Autocorrelation

While the bivariate relationships demonstrate directional improvements, evaluating spatial models against complex health outcomes requires controlling for socioeconomic confounders. To provide a more robust validation, we conducted a multivariate Ordinary Least Squares (OLS) regression predicting ‘poor or fair health’ using the revised accessibility scores while controlling for household income.

The multivariate regression (R-squared = 0.053) revealed that while income had the expected negative association with poor health (p = 0.065), the spatial accessibility score was not a statistically significant independent predictor (p = 0.494). Hospitals are heavily agglomerated in dense urban cores, yielding high spatial accessibility scores, yet these same environments frequently suffer from systemic, localized social determinants of health (e.g., extreme poverty, poor environmental quality) that override physical proximity to care. Furthermore, comparing highly granular block-group spatial access against aggregated county-level health outcomes likely introduces an ecological fallacy, washing out neighborhood-level effects. Finally, we assessed the distributional realism of the models using the Gini index and Global Moran’s I (Table 3).

**Table 3:** Quantitative comparison of spatial inequality and clustering.

Metric	Traditional E2SFCA	Revised E2SFCA
Gini Index (Spatial Inequality)	0.318	0.630
Global Moran's I (Spatial Clustering)	0.919	0.426
Moran's I p-value	0.001	0.001

The traditional model produced a highly smoothed accessibility landscape with low spatial inequality (Gini = 0.318) and extreme artificial clustering (Moran's I = 0.919). In contrast, the revised mobility-driven model unmasks significant disparities in actual healthcare accessibility (Gini = 0.630). While accessibility remains spatially clustered (Moran's I = 0.426), the clustering is far less artificial. These robust metrics confirm that the revised framework captures a much more granular and realistic landscape of healthcare access.

To further contextualize these validation results and explore the underlying mechanisms driving the massive variations observed in hospital catchment areas, we propose three distinct hypotheses for future research. First, facility specialization likely dictates catchment size: highly specialized facilities will exhibit expansive, exponential catchments, whereas general community hospitals will exhibit sharply bounded, power-law catchments. Second, the urban-rural spatial divide heavily influences mobility: rural hospitals will demonstrate geographically larger catchment areas due to the scarcity of alternative providers, whereas urban catchments will be smaller but more densely concentrated due to high facility competition. Finally, socioeconomic mobility barriers constrict travel behavior: catchment areas in low-income neighborhoods will exhibit steeper decay rates (restricted bounds) due to transportation inequities and limited vehicle access, compared to affluent areas where patients possess the resources to bypass local facilities for preferred care networks. Testing these hypotheses in future studies will be critical for unpacking the exact behavioral and structural determinants of spatial access.

## 6. Limitations

While this study advances the E2SFCA framework by incorporating large-scale human mobility data, it is subject to several limitations related to both conceptual scope and data constraints.

### 6.1. Conceptual limitations

First, while the revised E2SFCA model improves upon traditional assumptions by leveraging empirical mobility patterns, it does not fully address several non-spatial factors that critically shape healthcare access. These include financial and insurance-related barriers, linguistic or cultural compatibility, healthcare needs varying by population subgroups, and system-level constraints such as provider capacity, appointment availability, or wait times. Moreover, like most spatial models, the analysis assumes that individuals have perfect knowledge of all providers within their catchment area and base decisions primarily on distance, which oversimplifies the complexity of healthcare-seeking behavior. Although observed mobility patterns implicitly capture some aspects of patient preference, such as the tendency to bypass nearby facilities, this approach does not fully account for the nuanced reasons behind such choices. Modeling these dimensions would require detailed behavioral, demographic, and institutional data beyond the scope of this study.

Additionally, the model is cross-sectional in nature and does not account for temporal dynamics in healthcare accessibility. While the mobility data offers some time granularity, the supply-side data such as hospital capacity is static. This limits the ability to assess how accessibility changes in response to factors like seasonal demand surges, temporary hospital closures, or staffing fluctuations.

### 6.2. Data limitations

The SafeGraph (Advan) mobility data, while providing unprecedented spatial granularity, has inherent limitations regarding sample representativeness. It represents an anonymized panel of mobile devices covering approximately 7.5% of the population [10]. As demonstrated in recent comprehensive quantitative bias analyses, which evaluated distributional differences between SafeGraph mobility samples and ACS Census populations, the sampling is spatially and demographically uneven. Specifically, device location data systematically under-samples older adults (the elderly), low-income, low-education, and Hispanic populations [10]. Because these vulnerable demographic groups often face the highest barriers to healthcare access, their underrepresentation introduces potential biases and may lead to an overestimation of true accessibility in disadvantaged neighborhoods.

Furthermore, privacy protections and data aggregation result in the loss of granularity: routes are not traceable, and data are reported as aggregated flows or counts rather than individual-level trips. These limitations reduce the ability to infer exact travel paths, calculate precise travel times, or identify multimodal transport use (e.g., public transit versus driving), which is especially relevant for evaluating spatial access among underserved, transit-dependent populations.

## 7. Conclusion

This study enhances how we measure healthcare accessibility by bridging spatial theory with the complexities of real-world human behavior. By integrating human mobility data into the E2SFCA framework, we uncover inequities that traditional models fail to detect. A key contribution of this work is its ability to enhance the empirical validity of the E2SFCA model by improving its alignment with real-world patterns without adding the complexity of new behavioral or demographic parameters. This balance of accuracy and simplicity makes the revised model particularly appealing for policymakers seeking actionable tools.

Compared to the default E2SFCA, the revised model demonstrates stronger correlations with public health outcomes and household income, and reveals deeper disparities through a substantially higher Gini index. These results clearly highlight how healthcare is uneven and disconnected across different areas. Although this study does not explicitly integrate variables such as hospital popularity or individual patient preferences, it shows that these factors are implicitly captured through mobility-informed decay functions and dynamic catchments. In doing so, the revised model elevates patterns of patient choice and institutional draw that are otherwise invisible to traditional frameworks.

Looking ahead, this work offers insights into more dynamic, responsive models of healthcare accessibility where accessibility scores adapt in real time to flu outbreaks, emergency events, or temporary hospital closures. While data limitations such as sampling bias in mobility datasets remain, the findings suggest that spatial accessibility models should evolve from static approximations into living systems that reflect the fluidity of human lives and the shifting realities of care infrastructure.

## Supporting information

S1 File Socioeconomic and Income Data. Median household income at the Census Block Group level used to evaluate accessibility equity.
(CSV)

S2 File PA Hospital Capacity Data. Facility names, counties, and licensed bed counts used as a proxy for service capacity.
(CSV)

S3 File County-Level Health Outcomes. Percentage of the population reporting poor or fair health by county, used for model validation.
(CSV)

S4 File Anonymized Human Mobility Data. Aggregated visitor flows between Census Block Groups and Pennsylvania hospitals during 2023.
(CSV)

S5 TableHospital-level distance decay model calibration.Statistical fitting results including the selected functional form (power-law or exponential), β estimates, R², and AIC values.(CSV)
